# New measurement system for the spin correlation coefficients in deuteron–proton elastic scattering

**DOI:** 10.1140/epja/s10050-026-01902-8

**Published:** 2026-07-08

**Authors:** Y. Saito, K. Sekiguchi, A. Watanabe, K. Suzuki, H. Sugahara, D. Takahashi, K. Tateishi, S. Otsuka, H. Sakai, N. Sakamoto, T. Wakasa, H. Nishibata, K. Aradono, Y. Nagao, K. Hirasawa, Y. Maeda, H. Umetsu, J. Solà Cava, S. Heihoff, A. A. Filin, E. Epelbaum, J. Golak, R. Skibiński, H. Witała

**Affiliations:** 1 RIKEN Nishina Center, Wako, 351-0198 Japan; 2 Department of Physics, Institute of Science Tokyo, Tokyo, 152-8551 Japan; 3 Division of Physics and Astronomy, Kyoto University, Kyoto, 606-8501 Japan; 4 Graduate School of Science and Engineering, Saitama University, Saitama, 338-8570 Japan; 5 Department of Physics, Kyushu University, Fukuoka, 819-0395 Japan; 6 Quantum and Spacetime Research Institute, Kyushu University, Fukuoka, 819-0395 Japan; 7 Faculty of Education, University of Teacher Education Fukuoka, Fukuoka, 811-4192 Japan; 8 Faculty of Engineering, University of Miyazaki, Miyazaki, 889-2192 Japan; 9 Department of Physics, Tohoku University, Sendai, 980-8578 Japan; 10 Institut für Theoretische Physik II, Fakultät für Physik und Astronomie, Ruhr-Universität Bochum, D-44780 Bochum, Germany; 11 Faculty of Physics, Astronomy and Applied Computer Science, M. Smoluchowski Institute of Physics, Jagiellonian University, 30348 Kraków, Poland

## Abstract

Recent years have seen a growing need for high-precision data on spin observables in nucleon–deuteron (*Nd*) elastic scattering below the pion production threshold. Such data are expected to play a crucial role in advancing the understanding of the three-nucleon force (3*N*F) and in establishing its description within the framework of chiral effective field theory ($\chi$EFT). This paper presents a new measurement system developed at the RIKEN RI Beam Factory to address such demand, featuring a solid-state polarized proton target and the KuJyaku detector for deuteron–proton (*d*–*p*) elastic scattering experiments. In conjunction with the polarized deuteron beams provided by the polarized ion source at RIKEN, the system enables measurement of the spin correlation coefficients—for which data remain scarce—along with the deuteron and proton analyzing powers in *d*–*p* elastic scattering. Performances of the newly developed target and detector systems were evaluated under realistic beam conditions through deuteron–polarized proton scattering experiment at 135 MeV/nucleon. The angular distribution of the extracted relative differential cross-section and proton analyzing power exhibit close agreement with existing data, validating the identification of *d*–*p* elastic events using the KuJyaku detector. The absolute polarization of the solid-state polarized proton target was determined to be $0.034 \pm 0.004_\mathrm{stat.} \pm 0.001_\mathrm{sys.}$, with its stability under beam irradiations confirmed throughout the experiment. Discussions are presented on the estimated uncertainties of the spin correlation coefficients to be measured. The results are compared with the latest sensitivity studies on the low-energy constants in the 3*N*F sector of $\chi$EFT at the fifth order (N$^4$LO), demonstrating the feasibility of spin-observable measurements using the new experimental apparatus as a crucial step toward establishing the high-precision 3*N*F potential.

## Introduction

Necessity of the three-nucleon forces (3*N*Fs) has become evident in describing various nuclear phenomena, such as the nuclear binding energies [1, 2], the saturation density of nuclear matter [3, 4], and the existence limits of neutron-rich nuclei [5]. Initial theoretical treatments of 3*N*Fs, however, primarily focused on the two-pion-exchange type [6], leaving other possible components largely unexplored. In this context, recent theoretical developments based on chiral effective field theory ($\chi$EFT) have begun to systematically incorporate a broader range of 3*N*F diagrams, opening the way for a more quantitative and comprehensive understanding of the 3*N*Fs in modern nuclear physics.

Experimentally, properties of the 3*N*Fs have been investigated through few-nucleon scattering experiments, providing effective means for probing their momentum, spin, and isospin dependences. Direct comparisons between high-precision few-nucleon scattering data and theoretical predictions based on numerically exact solutions of the Faddeev equations offer detailed insights into the 3*N*Fs. Deuteron–proton (*d*–*p*) elastic scattering experiments, carried out between the 1990s and 2010s, yielded differential cross-section measurements at $\sim 100$ MeV/nucleon that clearly evidenced the presence of two-pion-exchange type 3*N*Fs [7–10]. At a higher energy of around 200 MeV/nucleon, however, discrepancies between data and theoretical calculations persisted even with the inclusion of conventional 3*N*F models, such as the Tucson-Melbourne [11, 12] and Urbana IX [13] potentials [9, 14, 15]. Furthermore, such 3*N*F models have shown limitations in describing data of the spin observables in *d*–*p* elastic scattering—namely the tensor and vector analyzing powers of the deuteron [7, 10, 16–19], analyzing power of the proton [9, 19, 20], the spin correlation coefficients [19], and the polarization transfer coefficients [10, 14, 21]. These findings indicate that the two-pion-exchange type 3*N*F alone is insufficient to reproduce observables in the high momentum transfer region, or to account for the spin-dependent components in the nuclear force.

In response to such limitations, the development of chiral effective field theory ($\chi$EFT)—based on low-energy quantum chromodynamics—marks a significant step forward, providing a systematically improvable framework for deriving nuclear interactions through an order-by-order expansion known as power counting [22]. Its two-nucleon (*NN*) force sector, incorporating interactions up to the fifth chiral order with selected higher-order contributions (N$^4$LO$^+$), has already achieved extremely high precision [23]. The resulting *NN* potentials outperform standard models such as the charge-dependent Bonn potential [24], the Argonne $v_{18}$ potential [25], and the Nijmegen I and II potentials [26] with greater precision in reproducing the available experimental data and fewer adjustable parameters, see [27] for details. They also exhibit small theoretical truncation uncertainties and minimal cut-off dependence. While applications of $\chi$EFT 3*N*Fs in three- and many-nucleon systems are currently limited to the third expansion order (N$^2$LO), a similar progress—after taking into account a wide range of diagrams at N$^3$LO and N$^4$LO—is expected to yield high-precision 3*N* potentials [28, 29].

Theoretical efforts are currently advancing toward the development of an accurate 3*N*F potential up to N$^4$LO, consistent with the latest $\chi$EFT *NN* interactions. Here, one significant challenge is related to regularization of the 3*N*F in the way compatible with the chiral symmetry [29]. To solve this problem, $\chi$EFT for few-nucleon systems was recently formulated using the symmetry-preserving gradient flow method [30, 31]. This rigorous approach is curren-tly being applied to derive consistently regularized N$^3$LO and N$^4$LO 3*N*F contributions. Once the complete expressions for the 3*N*F become available at the N$^4$LO accuracy level, one will need to determine various low-energy constants (LECs) entering the short-range part of the 3*N*F from experimental data. The dominant 3*N*F contributions at N$^2$LO depend on the LECs $c_D$ and $c_E$, which can be fixed, e.g., from the experimental values of the triton binding energy and the differential cross-section in *d*–*p* elastic scattering at 70 MeV/nucleon [1, 7], see also [32] for a related discussion. Additionally, thirteen LECs ($c_{E_i}$) appear in the purely short-range part of the 3*N*F at N$^4$LO [33, 34], whose determination is crucial for establishing the nuclear Hamiltonian at this order. By taking linear combinations of the thirteen independent short-range operators in the N$^4$LO 3*N*F, one finds eleven operators contributing in the isospin $T=1/2$ channel and two purely isospin $T=3/2$ contributions [34]. Accordingly, high-precision datasets of observables in nucleon–deuteron (*Nd*) scattering, limited to $T=1/2$, can in principle constrain eleven linear combinations of the $c_{E_i}$. Moreover, recent studies have demonstrated that spin observables in *Nd* elastic scattering below the pion production threshold exhibit pronounced sensitivity to the $c_{E_i}$, affirming their potential as powerful probes to pin down these LECs [28, 35]. Such high-precision data are thus in strong demand for the complete determination of the $\chi$EFT 3*N*F up to N$^4$LO—particularly at around 100 MeV/nucleon, where the truncation uncertainty of $\chi$EFT is still small and 3*N*F effects become prominent. Despite the demand, data remain scarce for certain spin observables [36, 37].

**Fig. 1:**
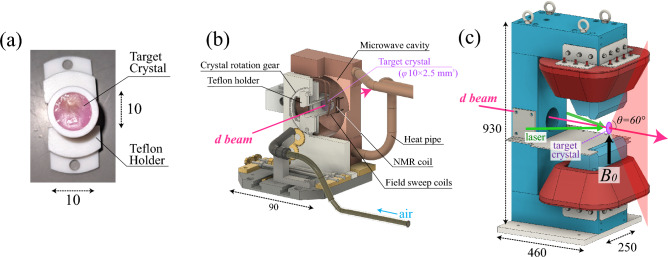
**a** Photograph of the $\phi$10 $\times$ 2.5 mm$^3$ *p*-terphenyl single crystal doped with 0.01 mol$\%$ pentacene-$d_{14}$, placed on a PTFE holder. **b** Schematic view of the target crystal mounted at the center of a TE$_{011}$ cylindrical microwave cavity equipped with magnetic field-sweep and NMR coils. **c** C-type electromagnet generating a magnetic field strength of 0.4 T at target position. The pole gap and coil geometry were designed to allow *d*–*p* measurements over an angular range of $|\theta _{\mathrm {lab.}}| \le 60^\circ$. All dimensions in mm

Motivated by the situation, in this paper, we present a new measurement system constructed at the RIKEN RI Beam Factory, enabling access to eight out of the twelve spin correlation coefficients in *d*–*p* elastic scattering at intermediate energies. The system also allows for measurements of the deuteron and proton analyzing powers at previously unmeasured incident energies and scattering angles, enabling simultaneous access to thirteen spin observables in *d*–*p* elastic scattering. The measurement system consists of the following components:

- Vector and tensor polarized deuteron beams generated by RIKEN’s polarized ion source [38].
- The solid-state polarized proton target, providing sufficient luminosity when operated in conjunction with the polarized or unpolarized deuteron beam at RIKEN.
- The KuJyaku detector, offering a wide angular acceptance covering the cross-section minimum region ($\theta _\textrm{CM}=70^\circ$–150$^\circ$ in the center-of-mass frame) at azimuthal angles $\phi = 0^\circ$, $90^\circ$, $180^\circ$, and $270^\circ$.

Notably, the solid-state polarized proton target and the KuJyaku detector were newly developed for this measurement. Using unpolarized deuteron beams, the two systems were deployed in a deuteron–polarized proton scattering experiment at 135 MeV/nucleon to confirm particle identification of the *d*–*p* elastic events using the KuJyaku detector, and to verify the stability and the absolute polarization of the new proton target.

The structure of this paper is as follows. Section 2 provides an overview of the new experimental apparatus designed to enable the measurement of spin correlation coefficients in *d*–*p* elastic scattering. Section 3 presents the experimental setup and analysis results from the deuteron–polarized proton scattering experiment described above. In Sect. 4, we discuss the estimated uncertainties of the spin correlation coefficients to be measured based on the performance of the new experimental apparatus, together with the latest sensitivity study results on the LECs in N$^4$LO 3*N*F. Finally, conclusions are drawn in Sect. 5.

## Experimental apparatus

### Polarized ion source

The polarized ion source at RIKEN has provided high-quality polarized deuteron beams, enabling precise measurements of deuteron analyzing powers over a wide energy range (70–300 MeV/nucleon) and exclusive observations of the polarization transfer coefficients in *d*–*p* elastic scattering [7, 16–18, 21, 39, 40]. In addition to achieving deuteron polarizations of 70–80% relative to the theoretical maxima for both vector and tensor components [17, 18], the system includes a spin-rotation Wien filter, enabling control of the deuteron polarization axis [41]. With the high-purity single-turn extraction feature of the azimuthally varying field (AVF) cyclotron and the RIKEN Ring cyclotron, polarized deuteron beams can be accelerated while maintaining their polarization for any orientation of the polarization axis. Such capabilities are essential for accessing the eight independent spin correlation coefficients in *d*–*p* elastic scattering.

The deuteron vector ($P_{Z}$) and tensor ($P_{ZZ}$) polarizations are defined in terms of the occupation probabilities $N_{+}$, $N_{0}$, and $N_{-}$ for the magnetic substates $m_{d} = +1$, 0, and $-1$, respectively, as:

1 $$\begin{aligned} P_{Z} = N_{+} - N_{-},~P_{ZZ} = 1 - 3N_{0}, \end{aligned}$$

in which the *Z*-direction is defined by the symmetry axis of the polarized ion source. Spin correlation coefficients will be measured using one unpolarized and three polarized modes, whose theoretical polarization maxima are given by $(P_{Z}, P_{ZZ}) = (0, 0)$, $(1/3, -1)$, $(-2/3, 0)$, and (1/3, 1). The four modes will be switched every 5 seconds to reduce systematic uncertainties. A beam test conducted in September 2024, marking the resumption of the ion source operation after its last operation in 2015, confirmed that the vector and tensor polarization could reach 70% of the theoretical maximum [42]. This performance provides a reliable basis for the forthcoming *d*–*p* experiments using the newly developed target and detector setups.

### Solid-state polarized proton target

A polarized proton system has been developed employing the triplet dynamic nuclear polarization (triplet-DNP) method [43, 44] for the forthcoming *d*–*p* elastic scattering measurements. The system satisfies the following conditions:

- a solid-state target providing sufficient luminosity when operated in conjunction with the deuteron beams at RIKEN ($\sim$1 nA),
- a magnetic field below 1 T to avoid significant deflection in the trajectories of scattered deuterons and recoil protons,
- an open aperture that allows detection of *d*–*p* elastic scattering over the cross-section minimum region ($|\theta _\mathrm{lab.}| \le 60^\circ$ in the laboratory frame).

Among the existing polarization methods, triplet-DNP uniquely satisfies all of the conditions above. This eliminates the need for high magnetic fields or cryogenic temperatures, enabling detections of scattered particles with relatively low kinetic energies ($\sim 15$ MeV/nucleon) while avoiding excess materials around the target crystal which would restrict the angular coverage. Details of the developed solid-state polarized proton target system are available in Refs. [45, 46].

Triplet-DNP based polarized targets have conventionally employed naphthalene single crystals as the host material, for applications to radioactive isotope beams [47–49] and neutron spin filters [50, 51]. In the present experiment, a *p*-terphenyl (C$_{18}$H$_{14}$) single crystal is employed for the first time for its potential resistance to polarization degradation under beam irradiation commonly observed in naphthalene targets [45, 49]. See Ref. [46] for experimental validation. A schematic view of the polarized proton target system is shown in Fig. 1. The $\phi 10 \times 2.5$ mm$^3$ *p*-terphenyl single crystal, doped with 0.01 mol% pentacene-$d_{14}$, is mounted on a Teflon holder, placed inside a microwave cavity, then positioned between the poles of a C-type electromagnet where a magnetic field of 0.4 T is applied. The shapes of the pole gap and coils are designed to allow measurements of *d*–*p* elastic scattering at $|\theta _\mathrm{lab.}| \le 60^\circ$, which corresponds to $\theta _\textrm{CM} = 57.8^\circ$–$180.0^\circ$ at 100 MeV/nucleon. In *d*–*p* scattering experiments, the trajectories of beam and scattered particles are affected by the magnetic field generated by the electromagnet. This effect is accounted for through trajectory simulations in combination with the newly constructed detector system, described in the following section.

### KuJyaku detector

The KuJyaku detector is designed to meet the following requirements for the *d*–*p* elastic scattering measurements:

- wide angular coverage including the cross-section minimum ($\theta _\textrm{CM}=70^\circ$–150$^\circ$),
- measurement at azimuthal angles of $\phi = 0^\circ$, $90^\circ$, $180^\circ$, and $270^\circ$,
- identification of *d*–*p* elastic events in kinematic coincidence to reduce background events,
- use of position-sensitive detectors to determine the scattering angles of deuterons and protons whose trajectories are affected by the magnetic field applied on target.

**Fig. 2:**
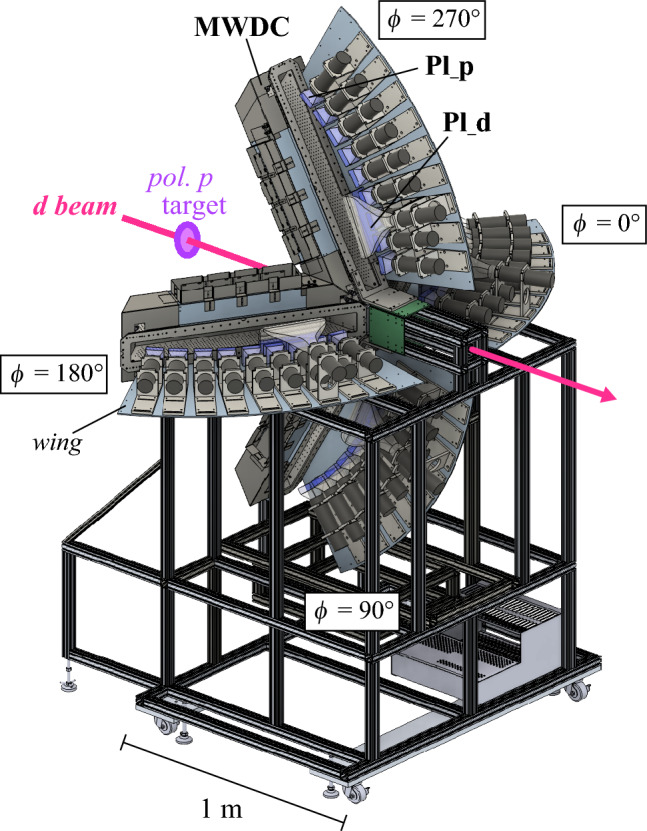
A schematic view of the KuJyaku detector. Four groups of detector sets are placed around the beam axis in the left, right, up and down directions, corresponding to the azimuthal angles of $\phi = 0^\circ , 180^\circ , 270^\circ$, and $90^\circ$, respectively

A schematic view of the KuJyaku detector is provided in Fig. 2. The KuJyaku is capable of measuring the *d*–*p* elastic scattering events at nine polar $\theta$ angles simultaneously. Four groups of detector sets, consisting of plastic scintillators and multi-wire drift chambers, are arranged around the beam axis in left, right, up, and down directions, corresponding to the azimuthal angles of $\phi = 0^\circ$, $180^\circ$, $270^\circ$, and $90^\circ$.

**Fig. 3:**
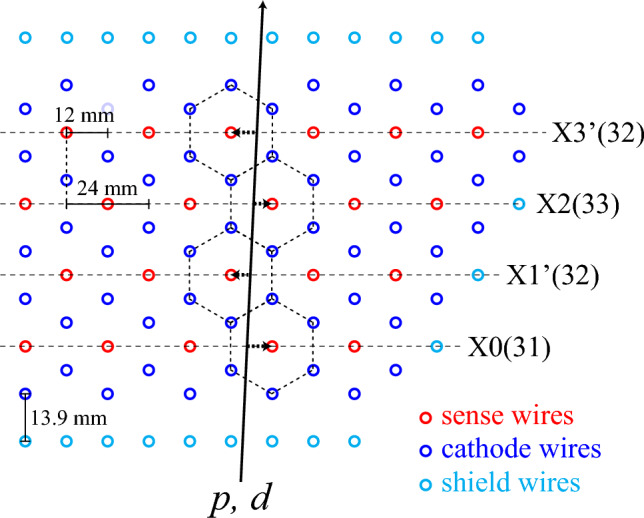
A schematic view of MWDC wire configuration

Scattered deuterons and recoil protons are detected under kinematic coincidence conditions using two types of plastic scintillators—the Pl$\_$d and Pl$\_$p detectors. The Pl$\_$d detectors ($250^{W} \times 70^{H} \times 10^{T}$ mm$^3$) are positioned 950 mm from the target center to detect scattered deuterons at $\theta _\mathrm{lab.} = 20^\circ$–$30^\circ$ and are required to coincide with one of the nine Pl$\_$p detectors ($70^{W} \times 70^{H} \times 25^{T}$ mm$^3$) placed on the opposite side of beam. The Pl$\_$p detectors are arranged 1000 mm from the target at five-degree intervals to detect recoiled protons at $\theta _\mathrm{lab.}=14^\circ$–$54^\circ$, corresponding to $\theta _\textrm{CM} = 69.6^\circ$–$150.8^\circ$ at 100 MeV/nucleon. This setup enables to reduce background events originating from carbon nuclei in the target crystal and deuteron breakups by utilizing the energy deposit and the time-of-flight difference between deuterons and protons.

To determine the optimal placement of the plastic scintillators, magnetic field and particle trajectory simulations were performed using TOSCA (OPERA-3d) [52] and GEANT4 [53], accounting for the 0.4 T field generated by the polarized proton target’s electromagnet. In *d*–*p* elastic scattering with incident energies of $\sim$100 MeV/nucleon, deuteron beams, as well as scattered deuterons and recoiled protons, are typically deflected by $2^\circ$–$4^\circ$. The scintillators are aligned along the simulated trajectories of deuterons and protons enabling detection of *d*–*p* elastic events at the desired scattering angles. For details on the magnetic field and trajectory calculations, see Ref. [45].

Multi-wire drift chambers (MWDCs) are installed in front of the plastic scintillators to validate the scattering angles and improve the spatial and angular resolutions of the detected deuterons and protons. The measured trajectories are directly compared with those from the simulations to evaluate the uncertainties in the scattering angles of the *d*–*p* elastic events. A schematic view of the MWDC wire configuration is shown in Fig. 3. The MWDCs have four *X*-planes (*X*0, $X1'$, *X*2, and $X3'$) with honeycomb structures consisting of sense wires ($\phi$30 $\upmu$m Au-plated W), and cathode and shield wires ($\phi$100 $\upmu$m Au-plated Be-Cu). The cell size is 24 mm, with *X*–$X'$ spacing of 12 mm. The numbers of sense wires in the respective planes are 31, 32, 33, and 32. The chambers are filled with a 50:50 gas mixture of argon and ethane.

**Table 1:** Present experimental conditions for the deuteron–polarized proton scattering measurement

Measured observables	$d\sigma /d\Omega$, $A_y^p$
Incident particle	Unpolarized deuteron
Incident energy	135 MeV/nucleon
Beam counting rates	10$^7$, $10^8$ counts per second
Target	*p*-Terphenyl single crystal (310 mg/cm$^2$)
Target polarization modes	Spin-up, spin-down, unpolarized
Detector	KuJyaku
Measured angles	$\theta _\textrm{CM}=68.8^\circ$–$150.3^\circ$

## Deuteron–polarized proton scattering experiment at 135 MeV/nucleon

### Experimental overview and setup

A deuteron–polarized proton scattering experiment was conducted at the RIKEN RI Beam Factory implementing the newly developed solid-state polarized proton target and the KuJyaku detector. The measurement had two primary objectives: (1) to validate the particle identification of *d*–*p* elastic scattering events detected with the KuJyaku, and (2) to confirm the absolute polarization of the proton target. To address objective (1), the relative differential cross-sections ($d\sigma /d\Omega$) and proton analyzing powers ($A_y^p$) were measured at $\theta _\textrm{CM} = 68.8^\circ$–$150.3^\circ$ in the center-of-mass frame, corresponding to KuJyaku’s angular coverage of $\theta _\mathrm{lab.} = 14^\circ$–$54^\circ$ at 135 MeV/nucleon. For objective (2), the absolute polarization of the proton target was determined from the measured yield asymmetry.

**Fig. 4:**
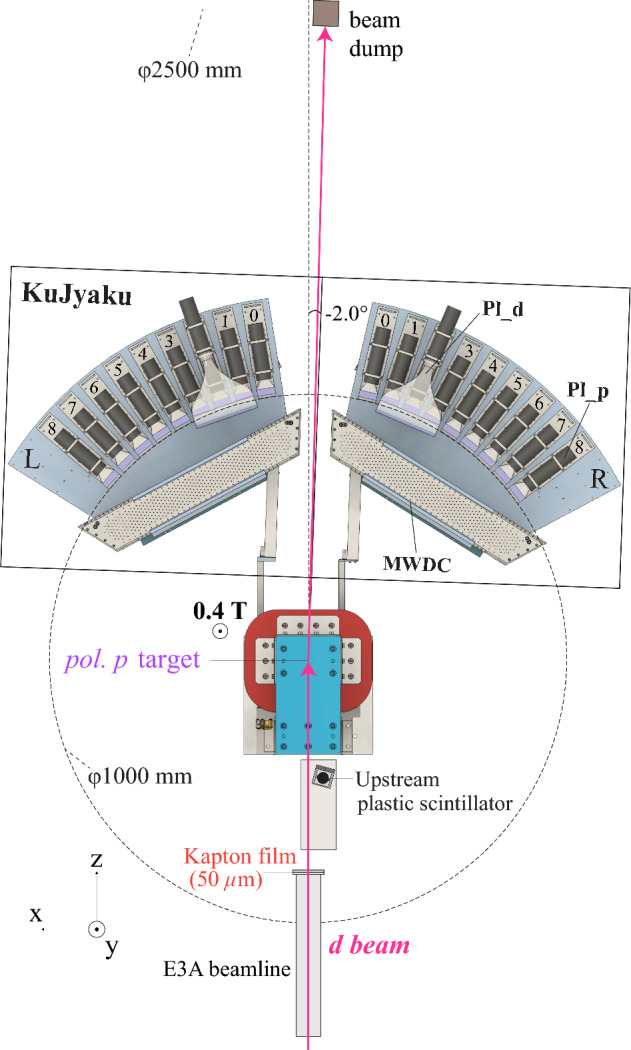
Top view of the experimental layout for the deuteron–polarized proton scattering experiment in the E3 experimental hall. The up and down wings of the KuJyaku detector are omitted for clarity

The experimental conditions are summarized in Table 1. The unpolarized deuteron beam provided by the electron cyclotron resonance ion source was first accelerated to 7 MeV/nucleon by the AVF cyclotron, and subsequently to 135 MeV/nucleon by the RIKEN Ring cyclotron. The accelerated beam was then transported to the E3A beamline in the E3 experimental hall, where the polarized proton target and the KuJyaku detector were installed at the downstream end of the beamline. A top view of the experimental layout, along with the definition of the coordinate axes, is shown in Fig. 4. The *z*-axis is aligned with the beam direction in the absence of the magnetic field, *y*-axis is directed vertically upward, and the *x*-axis is defined to form a right-handed coordinate system. The center of the target is taken as the origin. To avoid serious degradation of the target polarization due to beam irradiation, the beam counting rates were maintained at approximately $10^7$ and $10^8$ counts per second (cps). A plastic scintillator placed upstream of the polarized target with a 15$^\circ$ offset from the beam axis was used to monitor particles scattered from a 50 $\upmu$m-thick Kapton film. The proton target’s polarization was switched among spin-up, spin-down, and unpolarized modes during the experiment. Data taken in the unpolarized mode were used to determine the relative differential cross-section, while those taken in the polarized modes were used to extract the proton analyzing power and target polarization. The magnetic field of the polarized proton target was maintained throughout the experiment, including the unpolarized mode. To prevent the deflected deuteron beam from hitting the structural frames of the KuJyaku, and to ensure efficient particle detection by the detectors in upward and downward directions, the system was tilted by $-\,2.0^\circ$ from the *z*-axis in the *xz*-plane, i.e., rightward relative to the beam direction.

### Analysis

#### Event selection

The *d*–*p* elastic events collected by the KuJyaku detector were identified using the following information:

- time-of-flight difference between the Pl$\_$p and Pl$\_$d detectors,
- trajectories of deuterons and protons acquired by the MWDCs.

**Fig. 5:**
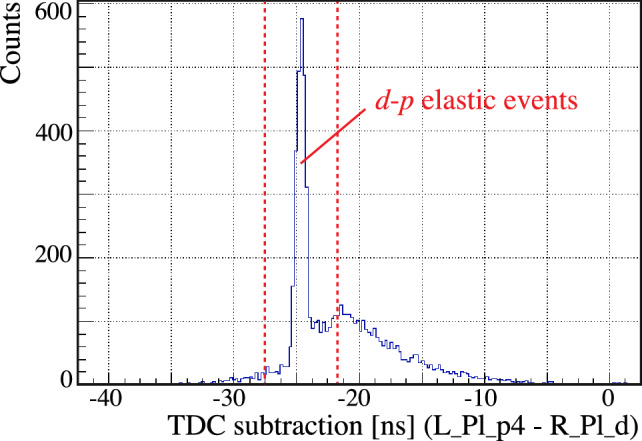
Time difference spectrum between the L$\_$Pl$\_$p4 and R$\_$Pl$\_$d detectors. A broad cut was applied to the time difference spectrum (between the red dashed lines), and the selected events were subjected to trajectory reconstruction using MWDC data

**Fig. 6:**
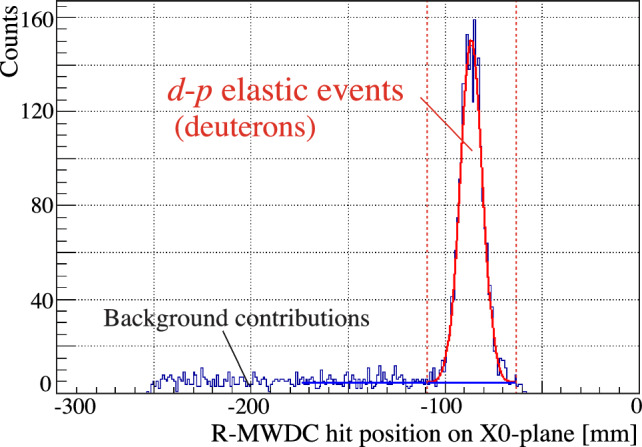
Hit positions on the *X*0-planes of R-MWDC, for events selected with the L$\_$Pl$\_$p4 and R$\_$Pl$\_$d detectors. Deuteron peak from *d*–*p* elastic scattering is clearly observed together with background contributions. The *d*–*p* elastic events were selected by fitting the peak with a Gaussian plus constant offset (shown with red and blue lines), and counting events within $\pm \,4\sigma$ (red dashed lines), with background contributions subtracted from the yield

Figure 5 shows a time difference spectrum between the L$\_$Pl$\_$p4 detector (the fifth left-side Pl$\_$p detector from the forward angles, set to detect recoil protons at $\theta _\mathrm{lab.} = 34^\circ$) and the R$\_$Pl$\_$d detector (the right-side Pl$\_$d detector). To suppress events induced by neutral particles, e.g., neutrons and $\gamma$ rays, the displayed data were filtered by requiring at least one hit in each of the four MWDC planes. The corresponding efficiencies were 96–99% at beam counting rates of $10^7$ cps and 91–98% at $10^8$ cps, depending on the measurement angle. A peak from *d*–*p* elastic events is clearly visible in the spectrum, along with background contributions originating from carbon nuclei in the target crystal and deuteron breakup reactions. A broad cut was applied to the time difference spectrum, and the selected events were subjected to trajectory reconstruction using MWDC data. The light output information of the plastic scintillators was not employed, so as to prevent undercounting of *d*–*p* elastic events that had undergone (1) nuclear reactions within the scintillators, or (2) passage through the detector edges, in particular for the extraction of the relative differential cross-section.

During the experiment, the MWDCs achieved position resolutions of 0.3–0.5 mm for protons and 0.3–0.4 mm for deuterons. The tracking efficiencies were 90–95% and 82–93% at beam counting rates of $10^{7}$ and $10^{8}$ cps, respectively, depending on the measurement angle. These efficiencies were evaluated from the fraction of events with trajectories reconstructed to the target position for both protons and deuterons. Figure 6 shows the hit positions on the *X*0-planes of right MWDC (R-MWDCs) for events selected by the L$\_$Pl$\_$p4 and R$\_$Pl$\_$d detectors, which shows a distinct deuteron peak from *d*–*p* elastic events accompanied by background contributions within acceptance of the R$\_$Pl$\_$d detector. The *d*–*p* elastic events were selected by fitting the peak with a Gaussian function plus a constant offset, and counting events within $\pm \, 4\sigma$. Background contributions beneath the elastic peak, estimated to be 6–14% according to the range of the TDC subtraction cut applied for each plastic scintillator, were subtracted from the yield. For the selected *d*–*p* elastic events, hit positions on the *X*0-planes and entry angles at the MWDCs were compared with the trajectory simulations, showing general consistency within $\pm \, 2$ mm and $\pm \, 0.2^{\circ }$. The residuals between the measured and simulated trajectories were used to estimate the angular uncertainties of each data set, yielding $|\Delta \theta _\textrm{CM}| \le 0.4^\circ$.

#### Extraction of relative differential cross-sections

The relative differential cross-sections were obtained from the experimental data using the following formula:

2 $$\begin{aligned} \frac{d\sigma }{d\Omega } = \frac{Y_{0}}{I \times n_t \times \Delta \Omega \times \epsilon _\mathrm{{det.}} \times \epsilon _\mathrm{{DAQ}}}, \end{aligned}$$

where $Y_{0}$ is the yield of *d*–*p* elastic events, *I* is the beam counting rate, $n_t$ is the target thickness, $\Delta \Omega$ is the solid angle of detectors, $\epsilon _\mathrm{{det.}}$ is the detection efficiency of the MWDCs, and $\epsilon _\mathrm{{DAQ}}$ is the DAQ live rate. The luminosity ($\mathcal {L} = I \times n_t$), a required input for this analysis, was determined using the detectors positioned in the up and down directions of the KuJyaku. The differential cross-sections were then evaluated with the calculated luminosity, using data obtained by the detectors placed in the left and right directions. Luminosities were obtained using Eq. (2) and experimental differential cross-section values from Refs. [7, 8], resulting in $(1.800\pm 0.004_\mathrm{stat.}\pm 0.031_\mathrm{sys.})\times 10^{29}$ cm$^{-2}$s$^{-1}$ and $(8.41\pm 0.02_\mathrm{stat.}\pm 0.14_\mathrm{sys.})\times 10^{29}$ cm$^{-2}$s$^{-1}$ at beam counting rates of $10^{7}$ and $10^{8}$ cps, respectively. The systematic uncertainties were determined as the quadratic sum of uncertainties arising from the interpolation of the data in Refs. [7, 8] and from the determination of the solid angle. Based on the geometry of the Pl_p detector, the solid angles of the detectors are typically 4.9 msr. In evaluating the associated uncertainty, effects from multiple scattering in the target crystal and air, geometrical constraints of the Pl_p and Pl_d detectors in the azimuthal direction, and asymmetries induced by the magnetic field were taken into account. The magnitudes of these effects were examined for detectors in the up and down directions using TOSCA and GEANT4 simulations, resulting in estimated uncertainties of less than 2%.

**Fig. 7:**
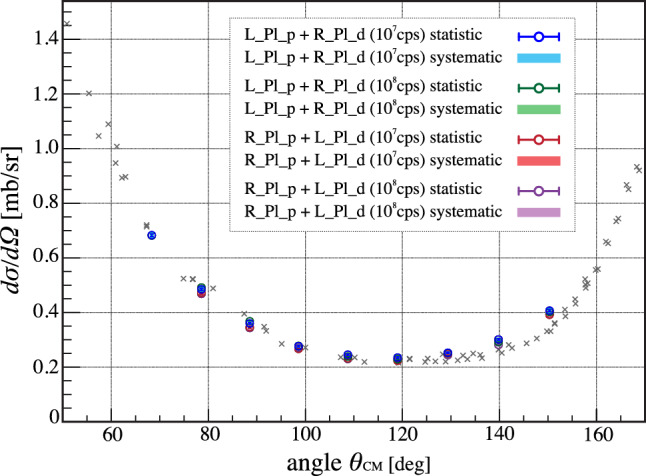
Experimental results of the relative differential cross-sections in *d*–*p* elastic scattering at 135 MeV/nucleon. Four data sets, obtained with the left and right-side detectors of KuJyaku at beam counting rates of $10^7$ and $10^8$ cps, are shown with statistical and systematic uncertainties. Data from Refs. [7, 8] are plotted with x-marks

Figure 7 shows the extracted differential cross-section values. Four data sets are presented, collected with the left and right-side detectors of the KuJyaku at beam counting rates of $10^7$ and $10^8$ cps, respectively. The statistical uncertainties at each data point were below 1.2%, while the systematic uncertainties ranged from 1.7–2.4%, obtained as the quadratic sum of the uncertainties from the luminosity and the solid angle. The uncertainties from the solid angles were evaluated using the same simulation procedure for the luminosity applied to the left and right-side detectors, resulting in uncertainties not exceeding 2%. The uncertainties in the measurement angles were $|\Delta \theta _\textrm{CM}| \le 0.4^\circ$ as described in Sect. 3.2.1. The extracted values in this work are consistent within the evaluated uncertainties. The angular distributions exhibit agreement with existing data, corresponding to an overall root-mean-square deviation of 10% [7, 8].^1^

#### Extraction of proton polarization and proton analyzing power

The absolute value of the proton target’s polarization was determined using data taken by the left and right-side detectors of KuJyaku at a beam counting rate of $10^7$ cps. The extracted value was subsequently used to obtain the proton analyzing power from data taken at $10^8$ cps. Such a procedure was feasible due to the high stability of the polarized proton target: the NMR signal attenuation remained minimal throughout the experiment, with the polarization values at the two intensities agreeing within 4% [46].

**Fig. 8:**
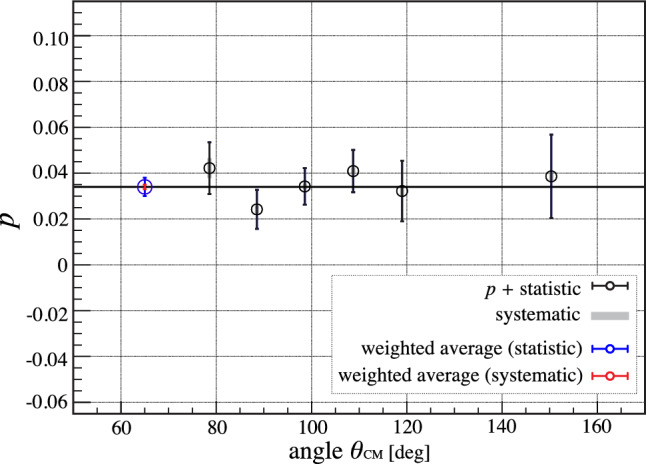
Proton polarization extracted using the left and right-side detectors of the KuJyaku. Polarization values were obtained at each measurement angle, and the weighted average was determined as the final result: $p = 0.034 \pm 0.004_\mathrm{stat.} \pm 0.001_\mathrm{sys.}$. Statistical (black/blue) and systematic (gray/red) uncertainties are shown for the values at each angle and for the weighted average, respectively

The yields measured by detectors placed at azimuthal angles $\phi = 0^\circ$ and $180^\circ$ with respect to the beam axis, corresponding to the left (*L*) and right (*R*) directions, respectively, for spin-up ($\uparrow$) and spin-down ($\downarrow$) proton polarization modes can be expressed as:

3 $$\begin{aligned} Y_L^{\uparrow } (\theta _L)= &   \sigma _0(\theta _L) I^{\uparrow } n_t \Delta \Omega _L \epsilon _{L\mathrm{det.}}^{\uparrow } \epsilon _\textrm{DAQ}^{\uparrow } \left( 1 + p^{\uparrow }A_y^p(\theta _L)\right) ,\nonumber \\ Y_L^{\downarrow } (\theta _L)= &   \sigma _0(\theta _L) I^{\downarrow } n_t \Delta \Omega _L \epsilon _{L\mathrm{det.}}^{\downarrow } \epsilon _\textrm{DAQ}^{\downarrow } \left( 1 - p^{\downarrow }A_y^p(\theta _L)\right) ,\nonumber \\ Y_R^{\uparrow } (\theta _R)= &   \sigma _0(\theta _R) I^{\uparrow } n_t \Delta \Omega _R \epsilon _{R\mathrm{det.}}^{\uparrow } \epsilon _\textrm{DAQ}^{\uparrow } \left( 1 - p^{\uparrow }A_y^p(\theta _R)\right) ,\nonumber \\ Y_R^{\downarrow } (\theta _R)= &   \sigma _0(\theta _R) I^{\downarrow } n_t \Delta \Omega _R \epsilon _{R\mathrm{det.}}^{\downarrow } \epsilon _\textrm{DAQ}^{\downarrow } \left( 1 + p^{\downarrow }A_y^p(\theta _R)\right) , \end{aligned}$$

where $\theta$ is the scattering angle, $\sigma _0$ is the unpolarized differential cross-section, *I* is the beam counting rate, $n_t$ is the target thickness, $\Delta \Omega$ is the solid angle, $\epsilon _\mathrm{{det.}}$ is the efficiency of the MWDCs, and $\epsilon _\mathrm{{DAQ}}$ is the DAQ live rate. *p* and $A_y^p(\theta )$ are the proton polarization and the proton analyzing power, respectively. When $\theta _L = \theta _R = \theta$, the following quantity $X(\theta )$ can be constructed from the yields in Eq. (3):

4 $$\begin{aligned} X(\theta ) = \frac{Y_L^{\uparrow }(\theta )}{Y_L^{\downarrow }(\theta )} \cdot \frac{Y_R^{\downarrow }(\theta )}{Y_R^{\uparrow }(\theta )} = \frac{1 + p^{\uparrow }A_y^p(\theta )}{1 - p^{\downarrow }A_y^p(\theta )} \cdot \frac{1 + p^{\downarrow }A_y^p(\theta )}{1 - p^{\uparrow }A_y^p(\theta )}. \end{aligned}$$

Note that $X(\theta )$ is independent of $\sigma _0(\theta )$, *I*, $n_t$, $\epsilon _\mathrm{det.}$, $\epsilon _\textrm{DAQ}$, and $\Delta \Omega$, all of which cancel out and therefore do not contribute to false asymmetries. In particular, asymmetries between the solid angles $\Delta \Omega _L$ and $\Delta \Omega _R$ are unavoidable, as the magnetic field applied to the target induces yield differences among the detectors. It is therefore essential that the spin observables, including the spin correlation coefficients to be measured, are extracted in a way to cancel out such false asymmetries. Provided that $p^{\uparrow }=p^{\downarrow }=p$, the proton polarization can be acquired as:

5 $$\begin{aligned} p = \frac{1}{A_y^p(\theta )} \frac{\sqrt{X(\theta )}-1}{\sqrt{X(\theta )}+1}. \end{aligned}$$

The experimental values from Refs. [19, 20] were used as inputs for $A_y^p(\theta )$. Polarization was first individually obtained at $\theta _\mathrm{lab.} = 14^\circ$, 29$^\circ$–$54^\circ$ as shown in Fig. 8, excluding data at $19^\circ$ and $24^\circ$ due to their small analyzing power values ($|A_y^p| < 0.15$). The systematic uncertainties were evaluated as the quadratic sums of two contributions: the confidence intervals of the reference $A_y^p$ values obtained via Legendre polynomial fits, and range in the input $A_y^p$ values from the angular uncertainty in the measurement. The final proton polarization, obtained as the weighted average of the individual values, was $p = 0.034 \pm 0.004_\mathrm{stat.} \pm 0.001_\mathrm{sys.}$.

**Fig. 9:**
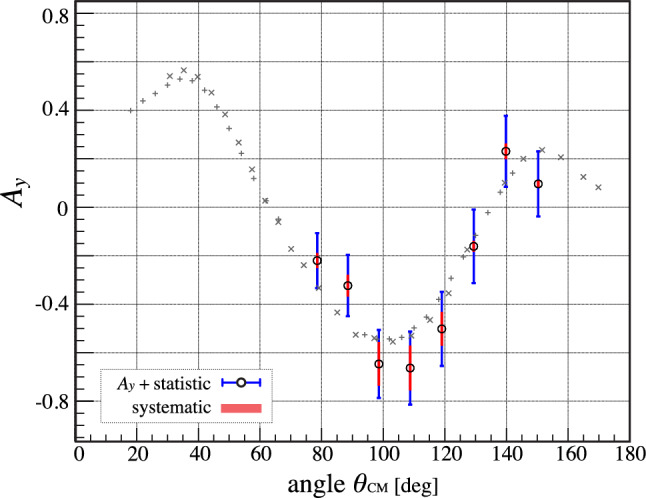
Experimental results of the proton analyzing power in *d*–*p* elastic scattering at 135 MeV/nucleon. Statistical and systematic uncertainties are shown in blue and red, respectively. Data by Refs. [19, 20] are shown with crosses and x-marks, respectively

Based on the extracted proton polarization (*p*), the proton analyzing power was obtained as:

6 $$\begin{aligned} A_y^p(\theta ) = \frac{1}{p} \cdot \frac{\sqrt{X(\theta )} - 1}{\sqrt{X(\theta )} + 1}. \end{aligned}$$

The results are presented in Fig. 9, where the systematic uncertainties arise from the polarization value *p*. The uncertainties in the measurement angles were $|\Delta \theta _\textrm{CM}| \le 0.4^\circ$ as discussed in Sect. 3.2.1. The sizable uncertainties of the extracted $A_y^p$ reflect the low proton polarization and the limited available beam time of 48 h; the statistical and systematic contributions are below 0.15 and 0.09, respectively. Nevertheless, its angular distribution agrees reasonably well with the existing data [19, 20].

**Table 2:** Assumed experimental conditions for the uncertainty estimation of spin correlation coefficients in *d*–*p* elastic scattering at 100 MeV/nucleon

Beam time	One week
Beam counting rate	$10^8$ cps
Target	*p*-Terphenyl single crystal (310 mg/cm$^2$)
Solid angle	4.9 msr
Deuteron beam polarization ($P_Z$ and $P_{ZZ}$)	70%
Proton target polarization ($p^T$)	10%
Cross-section $(d\sigma /d\Omega )_\mathrm{lab.}$	1.6–2.7 mb/sr

**Table 3:** Expected statistical and systematic uncertainties of the spin correlation coefficients $C_{y,y}$, $C_{yy,y}$, and $C_{x,x}$ and deuteron analyzing powers $A_{y}^d$ and $A_{yy}^d$ in *d*–*p* elastic scattering, evaluated under the experimental conditions listed in Table 2

	Statistical uncertainty	Systematic uncertainty
$C_{y,y}$	$\le 0.02$	$\le 0.05$
$C_{yy,y}$	$\le 0.02$	$\le 0.05$
$C_{x,x}$	$\le 0.02$	$\le 0.03$
$A_{y}^d$	$\le 0.01$	$\le 0.03$
$A_{yy}^d$	$\le 0.01$	$\le 0.03$

**Fig. 10:**
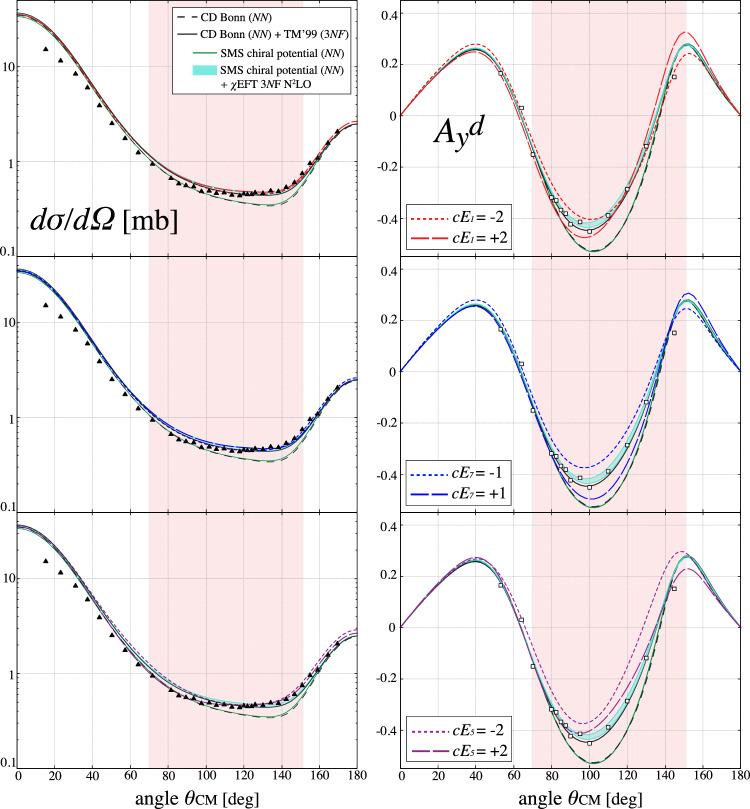
Exploratory studies on the effects the constants $c_{E_1}$, $c_{E_7}$ and $c_{E_5}$ in 3*N*F N$^4$LO on the differential cross-section and deuteron vector analyzing power $A_{y}^d$ in *d*–*p* elastic scattering at 100 MeV/nucleon. See the main text for notations. Data from Ref. [55] and Ref. [7] are shown with black triangles and white squares, respectively. The red shaded area indicates the angular coverage of the KuJyaku detector

**Fig. 11:**
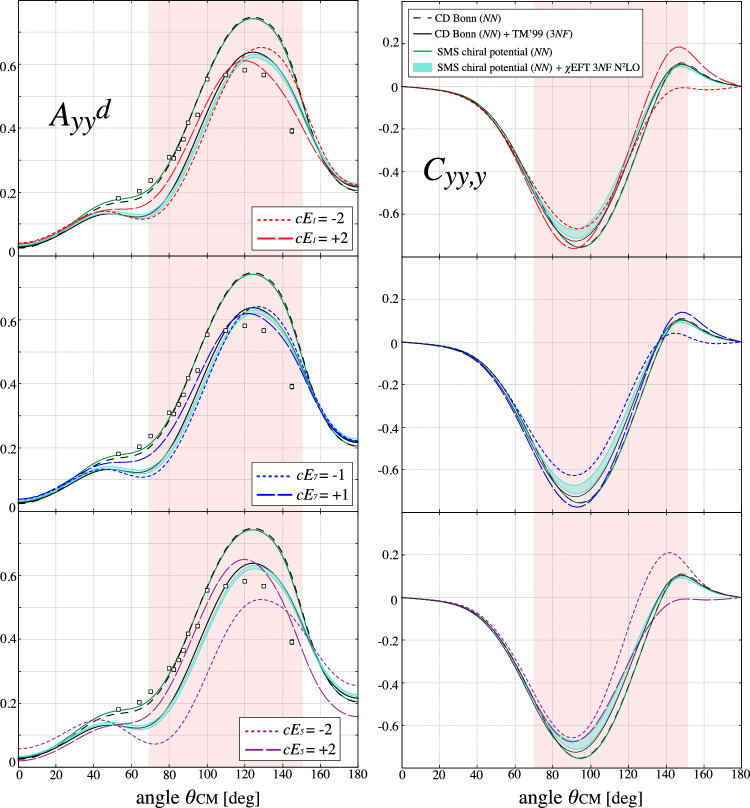
Exploratory studies on the effects of the constants $c_{E_1}$, $c_{E_7}$ and $c_{E_5}$ in 3*N*F N$^4$LO on the deuteron tensor analyzing power $A_{yy}^d$ and spin correlation coefficient $C_{yy,y}$ in *d*–*p* elastic scattering at 100 MeV/nucleon. Data from Ref. [7] are shown with white squares. See the main text for notations. The red shaded area indicates the angular coverage of the KuJyaku detector

## Estimated uncertainties of the spin correlation coefficients to be measured and expected sensitivities of the LECs in N$^4$LO 3*N*F

In the deuteron–polarized proton scattering experiment, the angular distribution of the extracted differential cross-section was found to be in agreement with existing data. Although the extracted analyzing powers involve sizable uncertainties, their angular distributions were also consistent with previous measurements. In conjunction with TOSCA and GEANT4 simulations, the uncertainties in the measurement angles of the scattered particles, affected by the 0.4 T magnetic field of the polarized proton target, were confirmed to be within $|\Delta \theta _\textrm{CM}| \le 0.4^\circ$. Taken together, these results demonstrate the successful identification of *d*–*p* elastic scattering events by the KuJyaku detector.

As for the polarized proton target, it was found to maintain consistent polarization throughout the experiment under beam irradiations up to $10^8$ cps [46]. Since the dominant systematic uncertainties in spin observables arise from target polarization, confirming its stability over the continuous operation was of crtical importance. Regarding the polarized ion source, its operational lifetime is known to be limited to about 1 week, which necessitates the accumulation of sufficient statistics within this period. In the measurements of the spin correlation coefficients, the absolute values of both beam and target polarization directly enter the denominator of the statistical uncertainties. With the deuteron beam polarization at 70% of its theoretical maximum, an increase in the proton target polarization of at least 10% is desirable. Accordingly, modifications to the optical system of the target apparatus are underway alongside the development of a new crystal employing *p*-terphenyl-$d_{4}$, with the aim of achieving higher polarization.

In light of the expected improvements, we consider the case where the measurement of spin correlation coefficients in *d*–*p* elastic scattering at 100 MeV/nucleon is carried out for 1 week under the conditions listed in Table 2. In this scenario, we evaluate the statistical and systematic uncertainties for $C_{y,y}$, $C_{yy,y}$, and $C_{x,x}$, three of the eight independent coefficients measurable with the KuJyaku detector, together with the simultaneously measurable deuteron analyzing powers $A_{y}^d$ and $A_{yy}^d$. The results are summarized in Table 3. The statistical and systematic uncertainties of the observables at each measurement angle are expected to be less than 0.02 and 0.05 for the spin correlation coefficients, and below 0.01 and 0.03 for the analyzing powers. The systematic uncertainties arise from the precisions of beam and target polarizations as inferred from prior experimental experience and present analysis: $\sim$ 3% for the deuteron vector and tensor polarizations [54], and $\sim$ 0.5% for the proton polarization.

**Fig. 12:**
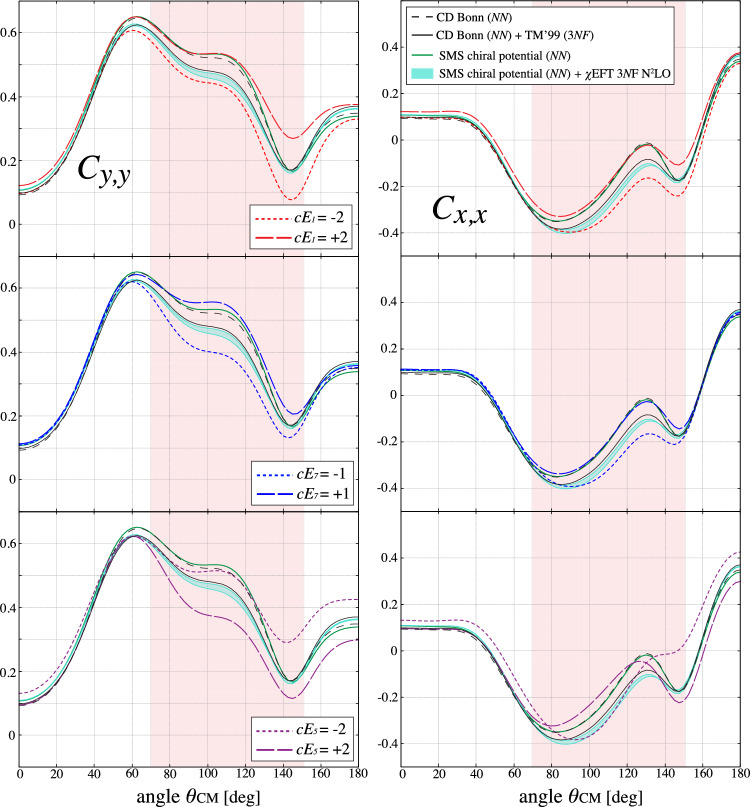
Exploratory studies on the effects of the constants $c_{E_1}$, $c_{E_7}$ and $c_{E_5}$ in 3*N*F N$^4$LO on the spin correlation coefficients $C_{y,y}$ and $C_{x,x}$ in *d*–*p* elastic scattering at 100 MeV/nucleon. See the main text for notations. The red shaded area indicates the angular coverage of the KuJyaku detector

Next, we demonstrate theoretical predictions for observables in *d*–*p* elastic scattering using the state-of-the-art *NN* and 3*N*F potentials. Figures 10, 11 and 12 present calculations for the differential cross-section, the deuteron analyzing powers $A_y^d$ and $A_{yy}^d$, and the spin correlation coefficients $C_{y,y}$, $C_{yy,y}$, and $C_{x,x}$ at 100 MeV/nucleon together with available data [7, 55]. The results are obtained from numerically exact solutions of the Faddeev equations without taking the coulomb interaction into account [56, 57]. The black solid and dashed lines indicate results using the standard CD-Bonn potential [24] with or without the TM’99 3*N*F [12]. The green solid lines show predictions of the state-of-the-art $\chi$EFT-based *NN* interactions, namely the semilocal momentum-space regularized (SMS) *NN* potential at the highest available order N$^4$LO$^+$ using the cutoff value of $\Lambda = 450$ MeV [23]. The cyan bands show the results based on the same SMS $\chi$EFT *NN* potential in combination with the $\chi$EFT 3*N*F at N$^2$LO for the same cutoff value [58].

The band width reflects the variation of the low-energy constant (LEC) $c_D$ in the 3*N*F sector ranging between 2 and 4, while $c_E$ is constrained by the triton binding energy. The two calculations employing the *NN* and 3*N* interactions are found to be nearly identical for all four observables, as are the results including the *NN* interactions alone. The consistency can be understood in light of the statement in Ref. [35]: “The chiral 3*N*F at N$^2$LO, which contains a two-pion-exchange parameter-free component supplemented by two contact terms, is more or less equivalent to the commonly used Urbana IX or TM99 3*N*Fs.” The agreement between theoretical and experimental results for the differential cross-section clearly shows the 3*N*F effect, whereas the deuteron analyzing powers, representing the spin observables in general, indicate that the present 3*N* interactions do not fully reproduce the experimental data. In particular, the calculations and data do not agree over the measured angular range of $A_{yy}^d$. This highlights the need for higher-order contributions in the 3*N*F.

To explore the impacts of higher-order contributions in $\chi$EFT 3*N*Fs, sensitivity analysis is performed in which the effects of every purely short-range structure in the 3*N*Fs at N$^4$LO are considered. To this end, a set of calculations was performed by setting one of the corresponding LECs $c_{E_i}$ with $i = 1, \ldots , 13$ to fixed values of $\pm \, 1$ or $\pm \, 2$. In each case, the LECs $c_D$ and $c_E$ entering the N$^2$LO 3*N*Fs have been fixed to reproduce the triton binding energy and the experimental data for the differential cross-section at 70 MeV/nucleon around its minimum [7], see Ref. [59] for details. Illustrative results are presented for $c_{E_1}$, $c_{E_7}$, and $c_{E_5}$. In Figs. 10, 11 and 12, the red, blue, and purple lines show the impacts from the inclusion of $c_{E_1}$, $c_{E_7}$, and $c_{E_5}$, respectively. For $c_{E_1}$, the red short- and long-dashed lines correspond to the calculation results with values set to $-\, 2$ and 2. Similarly, for $c_{E_7}$, the blue short- and long-dashed lines represent the results with values of $-\, 1$ and 1, while for $c_{E_5}$, the purple short- and long-dashed lines indicate the results for values of $-\, 2$ and 2. Note that the figures do not show the truncation uncertainties. The inclusion of $c_{E_i}$ has a minimal effect on the differential cross-section. In contrast, $c_{E_1}$, $c_{E_7}$, and $c_{E_5}$ have pronounced impacts of 0.1–0.2 on the spin correlation coefficients and deuteron analyzing powers in the cross-section minimum region, accessible by the KuJyaku detector. Such impacts are large enough to be resolved by high-precision measurements, given the estimated experimental uncertainty of $\sim$ 0.05. In this context, well-constrained data on a variety spin observables including the spin correlation coefficients, sensitive to the $c_{E_i}$ at N$^4$LO 3*N*F, are indispensable for determining the full set of $c_{E_i}$. The experimental system we have constructed at RIKEN—consisting of the polarized deuteron beam, the solid-state polarized proton target, and the KuJyaku detector—is an unparalleled setup capable of measuring eight spin correlation coefficients together with deuteron and proton analyzing powers, thus enabling simultaneous access to thirteen independent spin observables in *d*–*p* elastic scattering. The high-precision data set of the spin observables to be measured by the new system is expected to constrain the $c_{E_i}$ at N$^4$LO 3*N*F, thereby advancing the establishment and understanding of the 3*N*F. It may also help to determine LECs that appear in the one-pion-exchange-contact 3*N*F topology at N$^4$LO [60].

## Conclusion

A new measurement system has been developed at the RIKEN RI Beam Factory for precise measurements of the spin correlation coefficients in *d*–*p* elastic scattering to investigate the 3*N*Fs. The system features the polarized deuteron beams provided by the polarized ion source, a solid-state polarized proton target, and the KuJyaku detector. The newly developed target and detector systems were operated under realistic beam conditions in a deuteron–polarized proton scattering experiment at 135 MeV/nucleon, where angular distributions of the extracted relative differential cross-section and the proton analyzing power showed agreement with existing data, and thereby validating the identification of *d*–*p* elastic events using the KuJyaku detector. The absolute polarization of the solid-state polarized proton target was determined as $0.034 \pm 0.004_\mathrm{stat.} \pm 0.001_\mathrm{sys.}$, which was confirmed to be stable under beam irradiation. Based on the performance of the new experimental apparatus, the uncertainties of the spin observables to be measured are estimated to be typically around 0.05, whereas the latest sensitivity studies on the LECs at N$^4$LO 3*N*F in $\chi$EFT show that the spin observables exhibit sizable sensitivities of 0.1–0.2 in the cross-section minimum region. By realizing the estimated precision, the new experimental system at RIKEN is expected to yield first data on the eight spin correlation coefficients in *d*–*p* elastic scattering at 100 MeV/nucleon along with deuteron and proton analyzing powers, and constrain the LECs at N$^4$LO 3*N*F. Through the combined efforts of experiment and theory, a deeper understanding of the 3*N*F and the establishment of its high-precision potential should not be far in the future.

## Data Availability

Data will be made available on reasonable request. [Author’s comment: The datasets generated during and/or analysed during the current study are available from the corresponding author on reasonable request.]

## Footnotes

1 The differential cross-section was also normalized to data from Ref. [9], where the extracted angular distributions and data were found to be in agreement with an overall root-mean-square deviation of 9%.

## Code Availability Statement

Code/software will be made available on reasonable request. [Author’s comment: The code/software generated during and/or analysed during the current study is available from the corresponding author on reasonable request.]

## References

[1] E. Epelbaum et al., Phys. Rev. C **99**, 024313 (2019)

[2] S.C. Pieper, V.R. Pandharipande, R.B. Wiringa, J. Carlson, Phys. Rev. C **64**, 014001 (2001)

[3] K. Hebeler et al., Phys. Rev. C **83**, 031301(R) (2011)

[4] A. Akmal, V.R. Pandharipande, D.G. Ravenhall, Phys. Rev. C **58**, 1804 (1998)

[5] T. Otsuka, T. Suzuki, J.D. Holt, A. Schwenk, Y. Akaishi, Phys. Rev. Lett. **105**, 032501 (2010) 20867759 10.1103/PhysRevLett.105.032501

[6] J. Fujita, H. Miyazawa, Prog. Theor. Phys. **17**, 360–365 (1957)

[7] K. Sekiguchi et al., Phys. Rev. C **65**, 034003 (2002)

[8] K. Sekiguchi et al., Phys. Rev. Lett. **95**, 162301 (2005) 16241788 10.1103/PhysRevLett.95.162301

[9] K. Ermisch et al., Phys. Rev. C **71**, 064004 (2005)

[10] H.R. Amir-Ahmadi et al., Phys. Rev. C **75**, 041001(R) (2007)

[11] S.A. Coon, M.D. Scadron, P.C. McNamee, B.R. Barrett, D.W.E. Blatt, B.H.J. McKellar, Nucl. Phys. A **317**, 242–278 (1979)

[12] S.A. Coon, H.K. Han, Few-Body Syst. **30**, 131–141 (2001)

[13] B.S. Pudliner, V.R. Pandharipande, J. Carlson, S.C. Pieper, R.B. Wiringa, Phys. Rev. C **56**, 1720–1750 (1997)

[14] K. Hatanaka et al., Phys. Rev. C **66**, 044002 (2002)

[15] Y. Maeda et al., Phys. Rev. C **76**, 014004 (2007)

[16] K. Sekiguchi et al., Phys. Rev. C **83**, 061001(R) (2011)

[17] K. Sekiguchi et al., Phys. Rev. C **89**, 064007 (2014)

[18] K. Sekiguchi et al., Phys. Rev. C **96**, 064001 (2017)

[19] B. Przewoski et al., Phys. Rev. C **74**, 064003 (2006)

[20] K. Ermisch et al., Phys. Rev. Lett. **86**, 5862 (2001)

[21] K. Sekiguchi et al., Phys. Rev. C **70**, 014001 (2004)

[22] E. Epelbaum, H.-W. Hammer, U.-G. Meißner, Rev. Mod. Phys. **81**, 1773 (2009)

[23] P. Reinert, H. Krebs, E. Epelbaum, Eur. Phys. J. A **54**, 86 (2018)

[24] R. Machleidt, Phys. Rev. C **63**, 024001 (2001)

[25] R.B. Wiringa, V.G.J. Stoks, R. Schiavilla, Phys. Rev. C **51**, 38–51 (1995) 10.1103/physrevc.51.38 9970037

[26] V.G.J. Stoks, R.A.M. Klomp, C.P.F. Terheggen, J.J. de Swart, Phys. Rev. C **49**, 2950–2962 (1994) 10.1103/physrevc.49.2950 9969572

[27] E. Epelbaum, H. Krebs, P. Reinert, Semi-local nuclear forces from chiral EFT: state-of-the-art and challenges, in *Handbook of Nuclear Physics*. ed. by I. Tanihata, H. Toki, T. Kajino (Springer, Singapore, 2023), pp. 1853–1877

[28] E. Epelbaum et al., Eur. Phys. J. A **56**, 92 (2020)

[29] E. Epelbaum, H. Krebs, P. Reinert, Front. Phys. **8**, 98 (2020)

[30] H. Krebs, E. Epelbaum, Phys. Rev. C **110**, 044003 (2024)

[31] H. Krebs, E. Epelbaum, Phys. Rev. C **110**, 044004 (2024)

[32] S. Wesolowski et al., Phys. Rev. C **104**, 064001 (2021)

[33] L. Girlanda, A. Kievsky, M. Viviani, Phys. Rev. C **84**, 014001 (2011)

[34] L. Girlanda, A. Kievsky, M. Viviani, Phys. Rev. C **102**, 019903(E) (2020)

[35] H. Witała, J. Golak, R. Skibiński, Phys. Rev. C **105**, 054004 (2022)

[36] N. Kalantar-Nayestanaki et al., Rep. Prog. Phys. **75**, 016301 (2012) 22790304 10.1088/0034-4885/75/1/016301

[37] K. Sekiguchi, PoS CD2018, 107 (2020)

[38] H. Okamura et al., AIP Conf. Proc. **293**, 84 (1994)

[39] N. Sakamoto et al., Phys. Lett. B **367**, 60–64 (1996)

[40] H. Sakai et al., Phys. Rev. Lett. **84**, 5288–5291 (2000) 10990925 10.1103/PhysRevLett.84.5288

[41] H. Okamura et al., AIP Conf. Proc. **343**, 123 (1995)

[42] H. Sugahara et al., RIKEN Accel. Prog. Rep. **58**, 069 (2025)

[43] D. Sloop, T. Yu, T. Lin, S. Weissman, J. Chem. Phys. **75**, 3746 (1981)

[44] A. Henstra, P. Dirksen, W.T. Wenckebach, Phys. Lett. A **134**, 134–136 (1988)

[45] A. Watanabe, Nucl. Instrum. Methods Phys. Res. A **1078**, 170562 (2025)

[46] K. Tateishi et al., Phys. Rev. Res. **8**, L022005 (2026)

[47] M. Hatano et al., Eur. Phys. J. A **25**, 255–258 (2005)

[48] T. Uesaka et al., Phys. Rev. C **82**, 021602 (2010)

[49] S. Sakaguchi et al., Phys. Rev. C **84**, 024604 (2011)

[50] M. Haag et al., Nucl. Instrum. Methods Phys. Res. A **678**, 91–97 (2012)

[51] S. Takada et al., Prog. Theor. Exp. Phys. **2020**(12), 123G01 (2020)

[52] J. Simkin, C. Trowbridge, IEE Proc. B **127**, 368 (1980)

[53] S. Agostinelli, Nucl. Instrum. Methods Phys. Res. A **506**, 250–303 (2003)

[54] K. Sekiguchi et al., Phys. Rev. C **79**, 054008 (2009)

[55] Y. Tameshige, Doctoral Dissertation, The University of Osaka (2008)

[56] W. Glöckle, H. Witała, D. Hüber, H. Kamada, J. Golak, Phys. Rep. **274**, 107 (1996)

[57] D. Hüber, H. Kamada, H. Witała, W. Glöckle, Acta Phys. Pol. B **28**, 1677 (1997)

[58] H. Witała, J. Golak, R. Skibiński, K. Topolnicki, E. Epelbaum, H. Krebs, P. Reinert, Phys. Rev. C **104**, 014002 (2021)

[59] P. Maris et al., Phys. Rev. C **103**, 054001 (2021)

[60] H. Huesmann, H. Krebs, E. Epelbaum, arXiv:2602.12879 [nucl-th]

